# Targeting the Metabolic Reprogramming That Controls Epithelial-to-Mesenchymal Transition in Aggressive Tumors

**DOI:** 10.3389/fonc.2017.00040

**Published:** 2017-03-14

**Authors:** Andrea Morandi, Maria Letizia Taddei, Paola Chiarugi, Elisa Giannoni

**Affiliations:** ^1^Department of Experimental and Clinical Biomedical Sciences, University of Florence, Florence, Italy; ^2^Department of Experimental and Clinical Medicine, University of Florence, Florence, Italy; ^3^Excellence Centre for Research, Transfer and High Education DenoTHE, University of Florence, Florence, Italy

**Keywords:** epithelial-to-mesenchymal transition, metabolic reprogramming, Warburg metabolism, OXPHOS, TCA cycle, oncometabolites, amino acid, lipids

## Abstract

The epithelial-to-mesenchymal transition (EMT) process allows the trans-differentiation of a cell with epithelial features into a cell with mesenchymal characteristics. This process has been reported to be a key priming event for tumor development and therefore EMT activation is now considered an established trait of malignancy. The transcriptional and epigenetic reprogramming that governs EMT has been extensively characterized and reviewed in the last decade. However, increasing evidence demonstrates a correlation between metabolic reprogramming and EMT execution. The aim of the current review is to gather the recent findings that illustrate this correlation to help deciphering whether metabolic changes are causative or just a bystander effect of EMT activation. The review is divided accordingly to the catabolic and anabolic pathways that characterize carbohydrate, aminoacid, and lipid metabolism. Moreover, at the end of each part, we have discussed a series of potential metabolic targets involved in EMT promotion and execution for which drugs are either available or that could be further investigated for therapeutic intervention.

## Introduction

**Figure 1:**
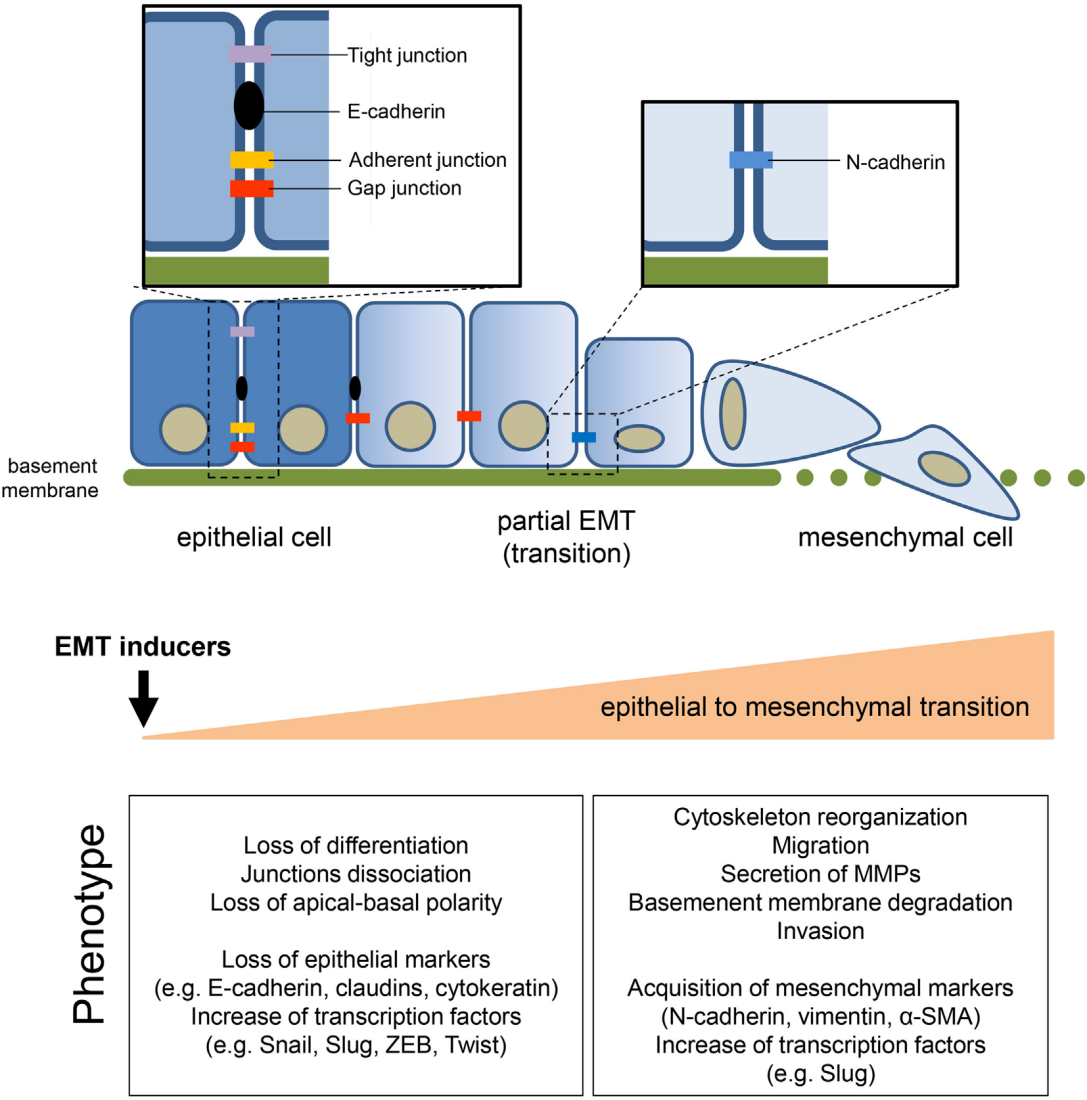
**General features of epithelial-to-mesenchymal transition (EMT)**. The transition of epithelial cells toward a mesenchymal phenotype, induced by several environmental or soluble factors, is characterized by the loss of cell–cell contact and cell polarity, which disrupts the epithelial architecture and endows the mesenchymal cells with migratory and invasive competences. EMT is accompanied by the modulation of well-known markers, among which the loss of epithelial marker E-cadherin, induced by the upregulation of its transcriptional repressors (i.e., Snail1/2, Twist, ZEB1/2), is one of the priming event. The concomitant acquisition of mesenchymal markers sustains and stabilizes the newly acquired phenotype.

The epithelial-to-mesenchymal transition (EMT) process allows the trans-differentiation of a cell with epithelial features into a cell with mesenchymal characteristics (Figure 1). This process has an essential role in physiological conditions (e.g., development, wound healing, and stem cell maintenance) and has been extensively reported to contribute pathologically to fibrosis and cancer progression. Interestingly, cells can also undergo the mesenchymal-to-epithelial transition (MET) process. MET is also essential in physiological conditions (e.g., during embryogenesis) but a key role in the formation of secondary metastases was reported (1, 2). The focus of the current review is on EMT in cancer progression although this process shares common characteristic with the physiological EMT program. The molecular mechanisms that an epithelial cell undergoes during EMT are tightly regulated and influenced by the cell-to-cell signaling network and by environmental factors. Independently of the stimuli that induce EMT, loss of E-cadherin is considered the key event and Snail1, Snail2 (also known as Slug), Twist, and ZEB1 are established transcription factors that can regulate E-cadherin expression. These mechanisms have been elegantly reviewed in Ref. (3). Conversely, the metabolic rewiring that sustains an epithelial cell undergoing EMT has been poorly investigated. However, since the metabolic reprogramming is now considered a hallmark of cancer (4), increasing evidence links metabolic deregulation to EMT program. This review gathers the recent findings on EMT and metabolic reprogramming in cancer and discusses how targeting certain metabolic pathways/hubs may impact on EMT and therefore on cancer progression.

## Metabolic Pathways that Controls EMT

### Targeting Carbohydrates Metabolic Reprogramming in EMT

Non-proliferating differentiated cells preferentially metabolize glucose to pyruvate through glycolysis and then oxidize this pyruvate *via* the tricarboxylic acid (TCA) cycle and subsequent oxidative phosphorylation (OXPHOS). This maximizes the efficiency of ATP generation from a single molecule of glucose. Otto Warburg first described that most cancer cells show increased glucose conversion into lactate even in oxygen-rich condition (aerobic glycolysis) (5). Aerobic glycolysis allow proliferating cell to satisfy three basic needs of cells in rapid division: (i) fast ATP; (ii) carbohydrates redirection to biosynthetic pathways; and (iii) cellular redox status homeostasis (6). As a direct consequences of a net increase in glucose consumption, many cancers exhibit abnormal lactic acid release and a more acidic extracellular pH (pHe) (7). Moreover, high level of lactate correlates with metastases of several types of human cancers (8). In this scenario, it is not surprising that hypoxia, low pHe, and glucose utilization are established features of many solid tumors and concur to EMT and cancer dissemination. Thus, interfering with the glycolytic pathway could impair EMT and subsequent tumor progression and could be a potential anticancer strategy. However, to our knowledge, none of glycolysis inhibitors that have shown promising results in preclinical models are currently used in the clinic.

Several glycolytic enzymes have been found associated with invadopodia structures, protrusions of the plasma membrane (PM) that have a major role in extracellular matrix (ECM) degradation, and metastasis (9). In addition, the glycolytic-derived ATP is the main source for cell survival during metastatic dissemination (10, 11). Several papers indeed show a strict correlation between transforming growth factor-β (TGF-β)-induced EMT, glycolytic switch, and repression of mitochondrial function (12, 13). Dong et al. have demonstrated that in breast cancer, loss of fructose-1,6-bisphosphatase together with the loss of E-cadherin promote cancer stem cell (CSC)-like properties and cancer cell dissemination by enhancing β-catenin signaling and EMT program. These events are concomitant with the induction of glycolysis, increase of glucose uptake, and inhibition of oxygen consumption (14). Additionally, EMT induction in breast cancer cells is paralleled by the expression of glucose transporters, lactate dehydrogenase (LDH), monocarboxylate transporters (MCTs), and glycogen phosphorylase isoforms, key players in sustaining enhanced aerobic glycolysis (15). Moreover, the acquisition of a malignant and chemo-resistant phenotype is associated with EMT and aerobic glycolysis in gastric cancer (16).

The importance of glycolysis for EMT is also reinforced by a metabolite profiling approach conducted in pancreatic ductal adenocarcinoma (PDAC) cells. This profiling identified three main subpopulations with different phenotypes: interestingly, the clone that is enriched for glycolytic-related metabolites is characterized by a mesenchymal phenotype. Since mesenchymal properties are positive correlated with cancer aggressiveness and diseases progression, the study reinforced the functional relevance of glycolytic metabolism in disease progression (17). In addition, the exposure of PDAC cells to established EMT inducers [i.e., tumor necrosis factor-α (TNF-α) and TGF-β] increases glucose uptake and lactate secretion, without affecting OXPHOS metabolism (18).

Glycolytic metabolism has been also associated to CSC, a phenotype that shares common molecular pathways with EMT, and characterizes the cells that within the tumor tissue are responsible for tumor repopulation, therapy resistance, and relapse in several cancer types (19). However, contradictory results described the CSC metabolic phenotype as glycolytic or OXPHOS addicted not only in various tumor types, but also within individual cancer types. Some studies have postulated that glycolysis supports the self-renewal ability of CSCs by maintaining low ROS levels (19, 20) and in line with this glucose deprivation reduces the number of CSCs in *in vitro* studies (21). In addition, the enhancement of the glycolytic flux has been shown to be paralleled by a decrease in mitochondrial metabolism (i.e., TCA cycle and OXPHOS) with respect to their differentiated counterparts (22–26). Gammon et al. demonstrated that the CSC fraction of head and neck squamous cell carcinoma that undergoes hypoxia-induced EMT is characterized by glycolysis, a reduction in the oxygen consumption rate and decreased mitochondrial mass and ROS levels (27). Notably, Dong et al. demonstrated the concomitant acquisition of CSC-like properties, EMT, and induction of glycolysis following fructose-bisphosphatase 1 silencing in breast cancer (14).

We now proceed analyzing the principal players involved in sustaining the Warburg metabolism with a particular focus on the transporters and glycolytic enzymes whose deregulation has been linked to the acquisition of EMT traits and subsequent enhanced metastatic potential in cancer cells.

#### Hypoxia

**Figure 2:**
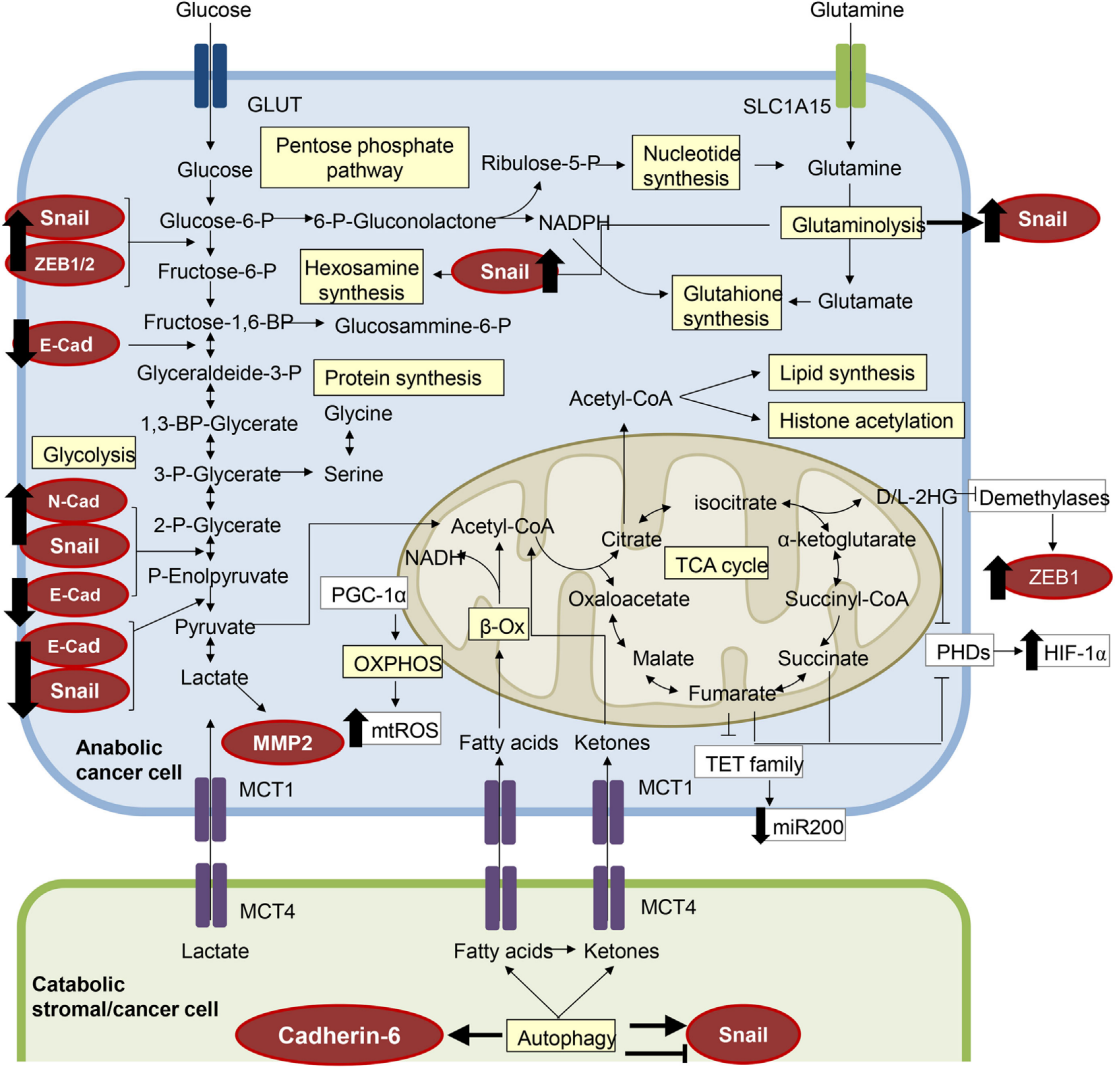
**The metabolic features of epithelial-to-mesenchymal transition (EMT)**. Several components of the molecular pathways driving EMT have a clear impact on cell metabolism and *vice versa*, resulting in a metabolic rewiring which sustains the EMT transcriptional program. EMT-committed cancer cells could rely on an aerobic glycolytic metabolism or could shift toward the more efficient oxidative phosphorylation (OXPHOS), dependent on tumor type and/or tumor stage. Here, we have highlighted in red circles/ellipses EMT transcriptional factors that are affected by the metabolites reported in different tumor types.

In fast-growing solid cancers, the inner zone of the tumor becomes progressively hypoxic and acid. These environmental inputs drive the expression and stabilization of the hypoxia-inducible factor-1α (HIF-1α). HIF-1α is responsible for the transcriptional activation of genes involved in the restoration of local oxygen perfusion and in the enhancement of the glycolytic flux (28, 29). In addition to its canonical role, HIF-1α is able to regulate the expression of matrix metalloproteases (MMPs) (30) and several hypoxia-inducible EMT-related genes (31) such as Snail1, Slug, and Twist. In addition, HIF-1α regulates the autocrine motility factor (AMF), the secreted form of the glycolytic enzyme glucose phosphate isomerase (PGI). AMF ectopic expression triggers EMT in breast cancer cells (32). Since hypoxia is a well-known inducer of EMT in epithelial cancers, such as PDAC (33), hepatocellular (34), ovarian (35), and lung cancers (36), we can speculate that this may be partially due to the metabolic reprogramming orchestrated by HIF-1α. More recently, also in glioblastoma, it has been demonstrated that HIF-1α silencing is able to prevent EMT induced by the hypoxic microenvironment (37). Studies from our laboratory have shown that cancer-associated fibroblasts (CAFs) promote EMT of prostate cancer cells through an ROS-dependent HIF-1α stabilization. This transition is paralleled by the acquisition of stem-like and metastatic traits of cancer cell (38, 39). In keeping, hypoxic senescent fibroblasts, which display a CAF-like phenotype with increased lactate production, are also capable of promoting EMT in prostate cancer cells (40). Due to its important roles, HIF-1α is a potential target for anticancer therapy. Among the molecules described to be able to target HIF-1α (41), the compounds BAY87-2243, an inhibitor of its activity and stability, and the antisense oligonucleotide EZN-2968 entered phase I clinical trials (42). Hypoxia induces EMT not only *via* the transcriptional regulation of HIF-1α-dependent EMT genes but also *via* epigenetic mechanism of gene regulation (Figure 2). Indeed, HIF-1α is able to increase the expression of ten–eleven translocation 1 (TET1), which catalyzes the conversion of 5-methylcytosine to 5-hydroxymethylcytosine, thus inducing DNA demethylation. Hypoxia-induced expression of TET1 promotes EMT by complexing with HIF-1α and HIF-1β and activating the transcriptional activation of HIF-dependent EMT genes (43, 44).

#### Glucose Transporters

The glucose transporters 1 (GLUT1) and GLUT3 are transcriptionally induced by HIF-1, thus coupling the hypoxia with increased glucose uptake and glycolysis (45). Interestingly, overexpression of GLUT1 increases MMP-2 expression/activity and invasiveness of cancer cell (46). Moreover, GLUT3 expression correlates with both EMT markers (i.e., vimentin, Snail, Slug, ZEB1, ZEB2, Twist1) and glucose uptake in non-small cell lung cancer (NSCLC) cells (47). In addition, enhanced GLUT1 and/or GLUT3 expression is associated with poor prognosis in several type of human tumors (48). Numerous efforts have been made to test the efficacy of GLUT inhibitors as anticancer drugs: WZB117 and the natural flavonoid silibinin are under preclinical studies even if to our knowledge no specific GLUT1 inhibitors have been identified.

#### Lactate

Since lactate is the by-product of Warburg-dependent metabolism and increase of glycolysis is reported in several carcinomas, high levels of lactate are positively correlate with cancer aggressiveness in several tumors (8, 49). In many types of tumor a significant proportion of pyruvate is reduced into lactate, a reaction catalyzed by LDH5. Elevated LDH5 expression correlates with unfavorable prognosis in many human tumors and silencing of LDH5 impairs tumor initiation and progression (50–53). According to the pivotal role exerted by lactate in the promotion of the metastatic process, lactate can induce the upregulation of TGF-β2 expression (54), which in turn induces a mesenchymal pro-migratory phenotype of glioblastoma cells and promotes MMP-2 activation, ECM remodeling, and metastasis formation (55). It has also been reported that lactate is able to induce the production of TGF-β1 *in vivo* (56). In keeping, it has been shown that lactate administration promotes *in vitro* migration of human breast carcinoma cells as well as experimental lung metastases in mice intraperitoneally injected with lactate (57). Lactate is also a sensor of NAD^+^/NADH ratio, which is crucial for activity of the sirtuins histone deacetylase, a class of NAD^+^-dependent enzymes that possess either mono-ADP-ribosyltransferase or deacetylase activity (58). High levels of NAD^+^/NADH ratio correlate with energy stress and promote sirtuins activity. Some studies show that SIRT1 induces both EMT and metastasis by suppressing E-cadherin transcription (59, 60). Furthermore, our group has recently shown that CAF-induced lactate production increases activity of SIRT1 with consequent deacetylation/activation of peroxisome proliferator-activated receptor gamma coactivator 1 alpha (PPARGC1A, also known as PGC-1α), a master regulator of mitochondrial biogenesis. This activation is crucial for the achievement of EMT. Accordingly, PGC-1α silencing abolishes invasion and EMT of prostate cancer cells. This evidence shows LDH as a possible therapeutic anticancer target. Several different classes of inhibitor have been synthesized, such as the gossypol derivative FX-11, compound GNE-140, galloflavin, and others (61), although none of these molecules are under consideration in the clinic.

#### Monocarboxylate Transporters

Key elements of the lactate shuttles are the MCTs, being MCT4 mainly involved in export of lactate and MCT1 in the uptake of this catabolite. Several studies demonstrated that silencing or inhibition of MCT1 and MCT4 are able to decrease the migration and invasion of several cancer cells (62–65). In particular, Fiaschi et al. have demonstrated that silencing of MCT1 and inhibition of MCT1-mediate lactate upload by prostate cancer cells impairs tumor growth and lung micrometastasis formation (66). Indeed, the blockade of lactate import and/or export is an interesting target for alternative therapeutic approaches. The α-cyano-4-hydroxycinnamate (CHC), that targets MCTs, has been used successfully in preclinical models without major toxicity *in vivo* (67–69). The AstraZeneca MCT1 inhibitor AZD3965 is in phase I/II clinical trials in the United Kingdom. Importantly, MCT4 silencing was shown to reduce tumor cell migration *in vitro* and *in vivo* (62) and the AstraZeneca MCT4 inhibitor (AZ93) has been reported as an highly efficient and likely selective compound that is currently used in preclinical studies.

#### Acidity

The regulation of lactate transport directly impacts on extracellular acidity and, together with the increase of the glycolytic flux lead to an unbalance in pH gradient across PM of cancer cells. Indeed, it is well established that tumor tissue are characterized by low pHe and the role of tumor acidity in cancer invasion and metastasis is well recognized (70). Indeed, low pHe leads to apoptosis of normal tissue at the periphery of the tumor while generating a positive selective pressure on cancer cells, selecting subclones that are resistant to acidic environment. Furthermore, acidification promotes angiogenesis through the enhanced expression of the vascular endothelial growth factor (VEGF) (71) and interleukin-8 (72) induces adherens junctions dissociation (73) and ECM degradation and remodeling (70, 74). Moreover, low pHe is crucial for the metastasization process providing secretion/activation of idrolases, such as catepsin (75) and MMPs (76). Acidic pHe was found to induce EMT in human melanoma cells (77) and in Lewis lung carcinoma cells (78). Accordingly, exposure of tumor cells to an acidic environment prior to tail vein injection increases lung colonization in a model of experimental metastasis (79).

Several molecules act as key players of cancer-associated extracellular acidification, such as the sodium-proton exchange 1 (NHE1), carbonic anhydrase IX (CAIX), the sodium bicarbonate transporter 1, and anion exchange 2. These molecules are possible targets for therapy focused on disruption of acid–base balance in cancer cells. In particular, CAIX, a membrane bound isoform of CA transcriptionally regulated by HIF-1α, induces extracellular acidification catalyzing the CO_2_ hydration and its overexpression is associated with increased metastasis and poor patients survival in several cancers (80). Fiaschi et al. have shown that activation of CAIX in CAFs leads to an extracellular acidification that enhances MMP-2 and MMP-9 secretion, thereby driving the stromal-induced EMT in prostate cancer cells. Moreover, both genetic silencing and pharmacological inhibition of CAIX are sufficient to impede EMT and CAF-induced invasion of prostate cancer cells (81). Furthermore, CAIX silencing in CAFs decreases prostate cancer cell tumor growth and lung micrometastasis formation, indicating the enzyme as an ideal target for anticancer therapy. At the moment, indisulam, an inhibitor of CAIX is tested in clinical trials for the treatment of NSCLC (82).

#### Glycolytic Enzymes

Glycolysis is finely regulated and articulated in 10 different steps and this could offer an array of potential targets to impair the EMT process. Moreover, the presence of several potential targets allows the possibility to interfere with more steps, either simultaneously or sequentially. Blocking EMT and metabolic reprogramming could therefore be a potential successful therapeutic approach. Hexokinase (HK2) is a HIF-1 target gene that controls the first rate-limiting step of glycolysis and is often overexpressed in cancer (83–86). It has been recently reported that breast CSCs showed increased HK2 expression and glycolytic rate. Notably, the glycolytic inhibitor, 2-deoxyglucose counteracts breast cancer cells undergoing EMT in a dose dependent fashion (87, 88). Furthermore, lonidamine that targets mitochondria-bound HK is now tested in phase II–III clinical trials (89). The HIF-1 dependent PGI/AMF (see above) acts as a pro-metastatic signaling molecule, due to its ability to promote tumor migration, invasion, and metastasis (90–92). Indeed, PGI/AMF overexpression leads to EMT achievement through NF-κB activation and increased expression of Snail1, ZEB1, and ZEB2 (Figure 2), downregulation of miR-200s with concomitant loss of E-cadherin (93). In keeping with these observations, high PGI levels in the serum positively correlate with metastases in colorectal, esophageal squamous cell, and lung tumors (94–96). However, although PGI has an established pro-tumorigenic role, to our knowledge, no selective inhibitors are available for preclinical investigation.

Another potential target of the glycolytic cascade is aldolase that converts the fructose-1,6-bisphosphate to glyceraldehyde-3-phosphate and dihydroxyacetone phosphate. The aldolase A isoenzyme is commonly overexpressed in various cancers (97, 98) and its upregulation has been reported to induce lung carcinoma cell migration and EMT by decreasing the expression of E-cadherin and concomitantly increasing those of fibronectin and vimentin (97).

In addition to the aforementioned glycolytic enzymes, enolase-1 has been recently proposed as a therapeutic target for gene-based therapy. The overexpression of enolase-1 increases glycolysis, proliferation, migration, and invasion in NSCLC cells, a process that is in part mediated by the regulation of EMT genes (99). Indeed, silencing of enolase-1 impaired EMT execution as shown by Snail1 and N-cadherin downregulation and the concomitant increase of E-cadherin expression (99). A role for enolase-1 in promoting migration and invasion has been described also in endometrial carcinoma (100). Small-molecule inhibitors of enolase are available but none of them has entered in the clinical practice (101). Finally, the pyruvate kinase (PK) enzyme has been recently emerged as an important player in tumor progression. PK catalyzes the dephosphorylation of phosphoenolpyruvate into pyruvate, resulting in ATP production. Differentiated cells primarily express the M1 isoform of the PK enzyme, whereas tumor cells often express the embryonic M2 isoform, which can be expressed as a tetramer, in its active form, or as a dimer with lower affinity for phosphoenolpyruvate. Dimeric PKM2 (102) acts as a transcriptional coactivator of HIF-1α in cancer cells, thus promoting glycolysis (103). Recently, our group demonstrated a peculiar role of PKM2 in inducing EMT in prostate cancer cells. Indeed, soluble factors secreted by CAF induce in prostate cancer the oxidation and phosphorylation of PKM2 prompting the nuclear translocation of the enzyme and its association with HIF-1α and the differentially expressed in chondrocyte-1, a transcriptional repressor, which downregulates miR-205. This transcriptional complex induces EMT execution (through the upregulation of ZEB2 and Snail1), as well as the metabolic rewiring of cancer cells toward OXPHOS metabolism (39). To further corroborate the link between PKM2 and EMT, Hamabe et al. recently demonstrated that EMT stimulates nuclear translocation of PKM2 and subsequent interaction with the TGF-β-induced factor homeobox 2, which promotes histone H3K9 deacetylation and the subsequent downregulation of E-cadherin expression (104). The PKM2 inhibitor TLN-232 was assessed for cancer therapy in a phase II clinical trial but despite some initial promising results the recruitment for a second phase II trial has halted for legal reasons in 2010 (105). Other efforts have been made to identify novel small-molecule inhibitors selective for PKM2 (106). However, emerging data suggest that, at least some tumors do not require PKM2 (107, 108), thus lowering the interest about the targeting of PKM2 in cancer.

Moreover, the switch between PKM1 and PKM2 couples glycolysis to pentose phosphate pathway (PPP), a metabolic pathway parallel to glycolysis that partially oxidizes glucose to pentoses and generates NADPH. The PPP has been described to support tumor cell proliferation (109) and to counteract ROS production, due to the generation of NADPH. Indeed, several studies show that PPP is necessary to handle the enhancement of ROS levels in stress condition, such as anchorage independent growth and anoikis (110, 111). Despite the PPP has a role in invasion, little is known about its contribution to EMT. Indeed, scanty data have shown that 6-phophogluconate dehydrogenase downregulation reduces *in vitro* migration of lung carcinoma cells (112) and high expression of the transketolase-like protein 1 isoform was positively correlated with invasion and metastasis of several carcinoma (113–116).

In addition, the hexosamine biosynthetic pathway (117), that accounts for 2–5% of total glucose metabolism and is intimately interconnected with the glycolytic pathway, shows a correlation with EMT. Indeed, glucose that enters the glycolytic pathway can be diverted to produce *O*-linked *N*-acetyl-glucosamine (*O*-GlcNAc), a molecule that has a signaling and structural role in the cells (117). It has been demonstrated that the addition of an *O*-GlcNAc motif at serine 112 prevented Snail1 O-phosphorylation-mediated degradation thus promoting its stabilization (118), thereby providing a direct molecular link between glucose metabolism and EMT.

### Impact of Amino Acids Metabolism on EMT and Targeting Approaches

Glucose dependency of fast-growing tumor is paralleled by the higher amino acids requirements of these aggressive cancers. Indeed, targeting amino acid metabolism is now considered a potential therapeutic approach in many cancer types (119). Particularly, the role of glutamine in cancer progression has been extensively investigated due to the fact that glutamine is an essential amino acid for proliferating tumor cells. Glutamine can enter the oxidative TCA cycle, contribute to reductive carboxylation, and alter the NADPH production and redox balance. Glutaminase (GLS) catabolized glutamine conversion into glutamate, which is then oxidized by glutamate dehydrogenase (GDH) into α-ketoglutarate (α-KG), an intermediate of the TCA cycle. Silencing of GLS1 or impairing glutamine metabolism were able to counteract the induction of EMT mediated by growth factors (e.g., TGF-β) in breast and colon cancers by increasing Snail1-targeting miRNA expression and hence impacting on *Snail* stability (Figure 2). Importantly, GLS1 silencing impaired tumor growth and metastases formation (120). Moreover, since GDH was reported to control tumor aggressiveness and therapy response (121), Liu and colleagues hypothesized a link between GDH expression/activity and EMT. Indeed, they reported an association of GDH overexpression with metastasis formation and poor prognosis in colorectal cancer patients. These effects were demonstrated to be dependent, at least *in vitro*, on the EMT induction mediated by GDH/STAT3 axis (122). However, Aguilar and colleagues reported a differential contribution of amino acid metabolism to EMT. Indeed, prostate cancer cells that were selected to display a stable EMT phenotype uncoupled to CSC behaviors were shown to (i) reduce the consumption rate of glutamine and other ketogenic amino acids (i.e., leucine, isoleucine, lysine, threonine, tyrosine, tryptophan, and phenylalanine) and (ii) be less sensitive to glutamine metabolism inhibition (123). The differences observed may be due to the fact that the model used was peculiar and CSC and EMT programs were uncoupled. Indeed, it is usually difficult to distinguish whether the metabolic alterations observed in CSC are dependent of EMT or CSC states, since recent studies have found that the acquisition of CSC properties may occur in cancer cells independently of EMT (124).

Drugs that target glutamine transport into the cell or the conversion to α-KG have been designed and tested. GLS inhibitors have shown positive results in preclinical models; bis-2-(5-phenylacetamido-1,2,4-thiadiazol-2-yl) ethyl sulfide impairs cancer cells growth *in vitro* and *in vivo* (125, 126), the GLS inhibitor CB-839 is effective against triple-negative breast cancer (TNBC) and hematological tumors in preclinical studies (127, 128) and is currently moved on to clinical trials (129). Therefore, targeting GLS could be an effective strategy to block the EMT process and therefore impair invasive abilities of aggressive cancer cells (130).

An alternative source of amino acids in cancer cell is due to degradation and recycling of cellular components (e.g., autophagy). A role for protein degradation in controlling EMT has been postulated recently by investigating how interfering with lysosomal cathepsin proteases (i.e., lysosomal-dependent protein turnover) affects TGF-β-induced EMT. Indeed, Kern and colleagues found increased lysosome activity during EMT of mammary epithelial cells derived from the MMTV-PyMT mouse model and showed that cathepsin inhibitor E64d was able to impair TGF-β-induced EMT and invasion (131). Moreover, Akalay and coworkers reported that tumor cells undergoing EMT-induced autophagy regulate target recognition and lysis of cytotoxic T lymphocytes and may be exploited for immunotherapeutic strategies to block immune escape (132). However, conflicting results revealed that liver-specific autophagy-deficient mice showed reduced expression levels of epithelial-related genes and increased of the mesenchymal related suggesting a direct link between autophagy and EMT. Indeed, induction of autophagy degrades Snail1 thus inhibiting the TGF-β-mediated EMT (133). This inverse correlation was also reported for glioblastoma cells (134). The diverging results may be ascribed to the different models used; however the link between autophagy and EMT seems to be conceivable. Recently, this connection has been further reinforced by the demonstration that cadherin-6 (Figure 2), a type 2 cadherin that marks cells that undergo EMT and correlates with metastatic ability in papillary thyroid cancers, restrains autophagy, and promotes reorganization of mitochondrial network (135). Autophagy targeting *via* hydroxychloroquine has now moved into clinical trials and a recent review (136) discusses how inducing or inhibiting autophagy can be achieved in preclinical models.

Finally, amino acid-related metabolic pathways control the EMT process *via* epigenetic modifications. Indeed, the methionine synthesis pathway plays a pivotal role in controlling promoters’ methylation and subsequent gene expression regulation. DNA methyltransferases and histone methyltransferases transfer a methyl group from the S-adenosyl methionine (SAM) to DNA or to positive charged amino acidic residues of histones with the formation of the by-product S-adenosyl homocysteine (SAH) (137). Since SAH inhibits DNA methyltransferases and histone methyltransferases, the ratio between SAM and SAH is important for regulating DNA and histone methylation (138). Threonine, glycine, and serine are the amino acids that sustain SAM generation through the folate metabolism (139). Threonine dehydrogenase converts threonine into glycine and acetyl-CoA and together with methionine adenosyl transferase are the key enzymes involved in SAM biosynthesis. Since methionine adenosyl transferase can be localized into the nucleus, it provides an effective system for chromatin-localized biosynthesis of metabolites to allow an adequate and well-organized regulation of the histone and DNA methylation processes. This mechanism can be exploited for targeting the epigenetic regulation of EMT-related genes. Indeed, SAM-competitive inhibitors and SAH hydrolase inhibitors have been shown to inhibit the methyltransferase EZH2 thus impairing the overall methylation histone status leading to transcription reactivation of silenced genes, such as the EMT-related gene E-cadherin (140, 141).

### Impact of Lipid Metabolism on EMT and Targeting Approaches

Despite glucose and glutamine alterations have been extensively investigated in the context of cancer metabolism, increasing evidence are now pointing to a pivotal role of alterations to lipid-associated pathways (142, 143). Indeed, proliferating cancer cells are characterized by increased lipids and cholesterol dependency, which can be fulfilled by either increasing the upload of exogenous lipids or by over-activating their endogenous synthesis. The excess of lipids in cancer cells are stored in lipid droplets (LDs) and high LDs and stored-cholesteryl ester content in tumors are now considered as hallmarks of cancer aggressiveness (144).

Lipids encompass a vast class of biomolecules of unique chemical structure. Among their multiple biological functions, they contribute to cell compartmentalization, cell signaling, protein trafficking, membrane organization, and energy storage and production. Despite their established role in regulating a variety of processes during cancer development (145–147), little is known about the impact of lipid composition and metabolism during EMT.

A lipidomic approach performed on prostate cancer cells that underwent EMT following TNFα treatment revealed a significant increase in the synthesis of triacylglicerols (TAGs), sustained by a concomitant upregulation of fatty acid synthase (FASN) (148). It is conceivable that the newly synthesized TAGs, stored into LDs, may act not only as a reservoir of energy, but also of fatty acids (FAs), which could serve for protein modification, cell membrane remodeling, and the generation of pro-tumorigenic signals which support EMT-induced cell motility. In keeping with the increase of FASN during EMT, administration of the FASN inhibitor osthole to the MCF7 breast cancer cells abolishes cell scattering, EMT execution, migration, and invasion induced by the hepatocyte growth factor (149). In addition, in breast cancer cells undergoing EMT an increased expression of FASN resulted in saturated FAs accumulation, which are then relocated to the cell membrane and regulate lipid rafts organization, resulting in the activation of the EMT-inducer VEGF/VEGFR2 signaling (150).

Although the above-described reports deal with a supporting role of FASN-driven lipogenesis for EMT and cell migration, opposing observations are also described in the literature. The exposure of NSCLC cells to the EMT-inducer TGF-β suppresses the transcription of enzymes involved in lipogenesis, including FASN and acetyl-CoA carboxylase (ACC) (151). These enzymes are controlled by the carbohydrate-responsive element-binding protein (ChREBP) and sterol regulatory element-binding proteins (SREBPs). Snail1 mediated the suppression of the lipogenic program by repressing SREBPs and ChREBP levels. In addition to a decreased lipogenesis, TGF-β-induced EMT also promoted higher oxygen consumption and elevated intracellular ATP. Indeed, decreased FAs synthesis could divert acetyl-CoA into the catabolic pathways, e.g., TCA cycle and OXPHOS, which efficiently yield high amount of energy to support the elevated cell migration required for metastatic spreading. In addition, an increased availability of acetyl-CoA could foster histone acetylation/activation of genes responsible for EMT engagement, i.e., the transcription factors ZEB1, ZEB2, and Slug; the mesenchymal markers vimentin, N-cadherin, and fibronectin; and the CSC markers Sox2 and Nanog (152). Notably, the expression of the lipogenic enzymes FASN and ACC are downregulated in circulating cancer cells, when compared to their primary cancer counterpart (153). Interestingly, this metabolic switch toward a catabolic signature is reversible upon withdrawal of EMT-inducing stimuli. The functional role of this metabolic reversion is that, when metastatic cells finally localize to secondary sites, they have the potential to re-activate FASN expression, switching their metabolism back to lipogenic pathways, in order to sustain rapid cell proliferation and metastatic colonies formation. The observation that decreased lipogenesis might stimulate cell invasion and metastases raises significant attention in considering FASN targeting and/or other lipogenic enzymes as potential therapeutic target for cancer treatment. Indeed, this therapeutic intervention, besides inducing a temporary cancer growth inhibition could, in the long-term, increase the risk of metastasis.

Another strategy used by cancer cells to improve FAs availability for anabolic and catabolic purposes, is to increase the expression of acyl-CoA synthetases (ACSLs), which converts the long-chain FAs into acyl-CoA, critical for phospholipids and TAGs synthesis, lipid modification of proteins as well as for FAs β-oxidation (154). In colorectal cancer cells, the simultaneous overexpression of ACSL1, ACSL4, and stearoyl-CoA desaturase-1 (SCD), the rate-limiting enzyme converting saturated FAs into monounsaturated FAs, induced EMT and increased cellular migration and invasion (155). Furthermore, the combination of low doses of pharmacological inhibitors of ACSL and SCD selectively reduces cell viability of chemotherapy resistant colorectal cells, with no effects on normal colonocytes. The clinical relevance of these findings is emphasized in colorectal patients with tumors displaying ACSL1/ACSL4/SCD simultaneous overexpression, that show a poorer outcome and a higher risk of relapse compared to other patients within the same clinico-pathological stage.

In addition to the above discussed increase in TAGs, a reduction of C16:0 ceramide levels was also associated with EMT (148, 156). A recent study documented that TGF-β-induced EMT in human breast epithelial cells downregulates the expression of ceramide synthase 6 (CerS6), which produces C16:0 ceramide, further metabolized to C16:0 sphingolipids, such as sphingomyelin and glycosphingolipids, both enriched in the PM microdomains (156). CerS6 mRNA and protein levels are also reduced in TNBC mesenchymal cells as compared with non-TNBC epithelial cells. Downregulation of CerS6 in TNBC patients correlates with increased PM fluidity, a required feature to foster cell motility and metastatic spreading. Hence, CerS6 is emerging as a novel EMT-regulated gene, whose reduction in cancer correlates with a mesenchymal phenotype and, in turn, with tumor aggressiveness and poor prognosis. Accordingly, it is predictable that in patients with aggressive TNBC characterized by CerS6 downregulation, increasing the levels of C16:0 ceramide could impair the metastatic potential of this aggressive breast cancer subtype.

Further evidence indicates that EMT is associated with changes in sphingolipids metabolism. Guan and colleagues reported that EMT is associated with the alteration of ganglioside (i.e., sialic acid-containing glycosphingolipids) metabolism in normal mouse mammary gland and bladder cancer (157). Gg4 is one of the most relevant ganglioside involved in EMT execution, whose content is reduced upon TGF-β-induced EMT. Gg4 was reported to physically interact with key epithelial cell molecules, such as E-cadherin and β-catenin, likely facilitating epithelial intercellular adhesion *via* stabilization of the E-cadherin/β-catenin complex at the cell surface (157). The modulation of ganglioside pattern upon EMT is related, at least in part, to the altered expression of genes encoding ganglioside metabolizing enzymes, which are under the control of different transcription factors involved in EMT (i.e., Snail1, Twist, ZEB1) (158–160). Alongside the role of gangliosides, an involvement of sphingosine-1-phosphate (S1P) in EMT has also been reported. In hepatocellular carcinoma, where high levels of S1P in the serum correlate with poor prognosis (161), S1P activates the PI3K/AKT signaling pathway, resulting in MMP-7 upregulation. MMP-7 mediates the shedding of syndecan-1, a transmembrane heparan sulfate proteoglycan that regulate cell–matrix interaction, with a consequent increase in TGF-β1 production, a well-established inducer of EMT (162). A role of S1P in the modulation of other MMPs which promote cell invasion, such as MMP-2 and MMP-9, has also been proposed (163, 164).

**Figure 3:**
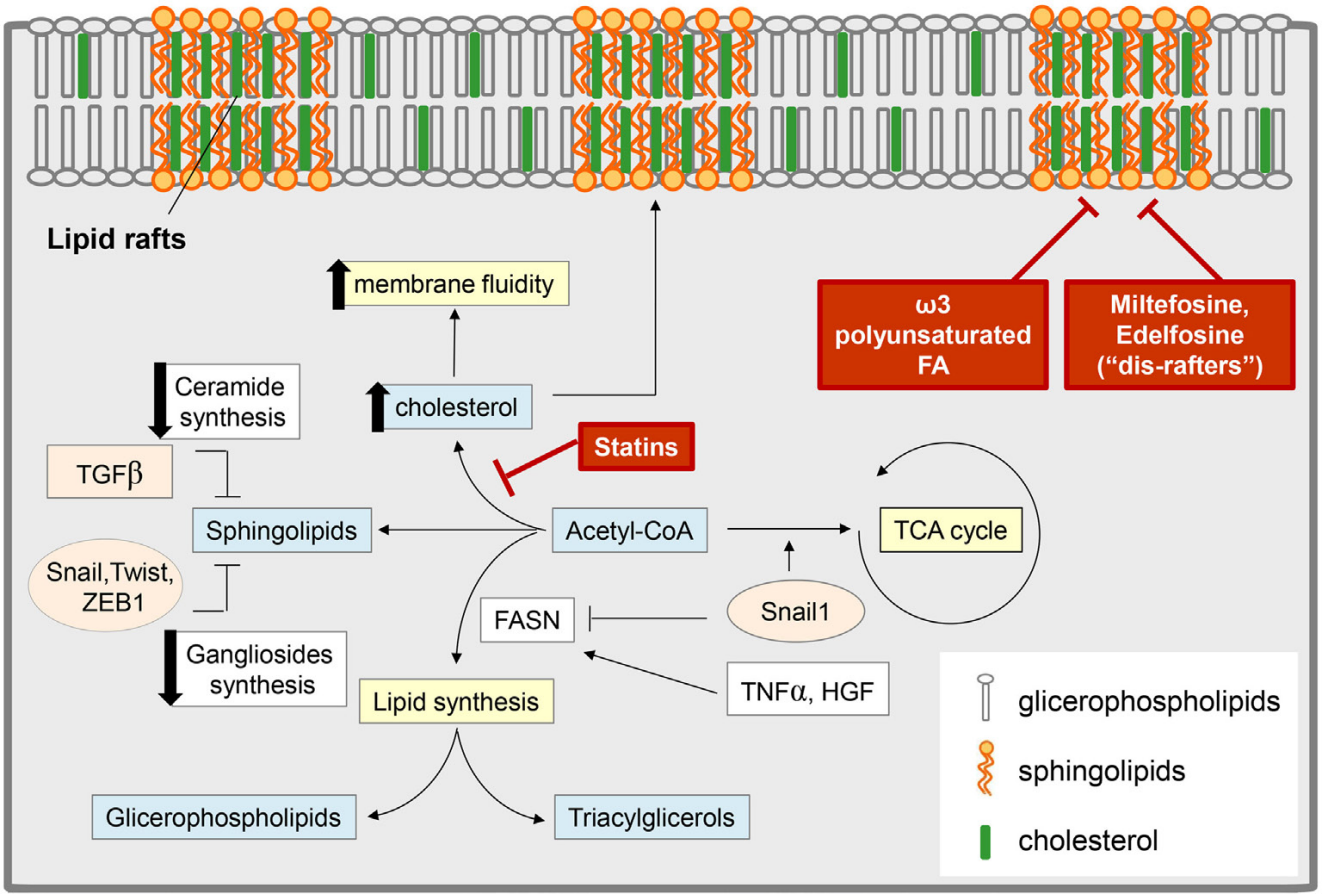
**Epithelial-to-mesenchymal transition (EMT) and lipid metabolism: interconnection and targeting**. Several EMT-induced stimuli modulate the content of different classes of lipids, with an impact on both cell metabolism and membrane composition. Some EMT inducers [i.e., tumor necrosis factor-α (TNF-α), hepatocyte growth factor (HGF)] promote lipogenesis by stimulating fatty acid synthase (FASN) and leading to an increase in glicerophospholipids (which are transferred to the cell membrane and regulate lipid rafts organization) and in triacylglycerol [stored in lipid drops as a reservoir of fatty acids (FAs) for catabolic or anabolic purpose]. On the other hand, other reports support a role for transforming growth factor-β (TGF-β)-derived Snail1 in the suppression of the lipogenic program by repressing FASN expression, thus diverting acetyl-CoA toward catabolic pathways, such as the tricarboxylic acid (TCA) cycle. TGF-β-induced EMT also correlates with a reduction in the pool of sphingolipids by (i) the downregulation of the expression of ceramide synthase, which results in the reduction of ceramide levels; (ii) the lowering of ganglioside content due to the control of EMT-related transcription factors (i.e., *SNAIL1, Twist, Zeb1*) on the expression of genes encoding ganglioside metabolizing enzymes. These events result in an increase of plasma membrane fluidity and destabilization of lipid rafts, to which significantly concurs also the upregulation of cholesterol content. Pharmacological inhibitors of cholesterol synthesis (i.e., statins), the administration of alkyl phospholipid drugs (the so-called “dis-rafters”), and nutritional factors, such as the ω-3 polyunsaturated FA docosahexaenoic acid, result in the stabilization of lipid raft and in the reduction of membrane fluidity, thereby counteracting EMT, invasion, and the acquisition of stem-like features.

In parallel with the acquisition of an aggressive phenotype, the EMT is marked by a profound rewiring of cell signaling pathways, most of them originating from PM located mediators (165, 166). It is therefore conceivable that alterations in the properties of the PM may impact on the overall signaling network rearrangements associated with EMT. Recent evidence showed that EMT is associated with a reorganization of the PM and in particular with a destabilization of lipid raft domains (Figure 3) (167). Alterations of the PM biophysical properties are required to maintain the mesenchymal state, associated with a CSC phenotype, and an increase in metastatic potential. Interestingly, stabilization of lipid rafts is emerging as a target for therapeutic strategies aimed to reduce the metastatic potential driven by EMT in cancer. The stabilization of lipid raft domains is a therapeutically attractive approach, since a number of pharmacological and nutritional factors have been shown to positively affect raft stability and function (168, 169). Among them, the essential ω-3 polyunsaturated FA docosahexaenoic acid (DHA) has been recently discovered to stabilize lipid raft and to antagonize both EMT and the acquisition of stem-like features (170). It suggests that the PM properties are subject to dietary inputs and that long-term perturbations in lipid metabolism (e.g., hypercholesterolemia, DHA supplementation) may profoundly affect tumor progression. In addition, alkylphospholipid drugs (i.e., miltefosine and edelfosine) that have been described as “dis-rafters” for their ability to affect raft organization have potent anti-neoplastic activity (169).

To reinforce the link between PM composition and EMT, alterations in membrane fluidity, e.g., by modulating cholesterol content (Figure 3), has been shown to induce or inhibit the conversion toward a mesenchymal phenotype (167). Indeed, the lipid compositions of epithelial or mesenchymal cells are distinct (171) and when a given cell undergo the EMT process it exhibits a significant increase in cholesterol content, which strongly contributes, together with the reduction in ceramide pool, to the enhancement in membrane fluidity. Accordingly, cancer cells undergoing EMT are more sensitive to cholesterol lowering drugs, such as statins, that have been reported to deplete cholesterol content in mesenchymal cells, thereby reducing PM fluidity and impairing cell motility and metastatic potential (172). Increased membrane fluidity is an emerging necessary feature of metastatic cancers that can be controlled by many currently available drugs, offering a feasible therapeutic opportunity to prevent cancer metastasis. A fascinating study has recently identified, using an *in silico* drug screening, a series of pharmacological compounds that can repress the metastatic phenotype of cancer cells by inhibiting a gene expression signature associated with EMT. The compounds discovered using this analysis, including previously acknowledged anti-metastatic drugs, appeared to restrict the metastatic capacity through a common mechanism, i.e., the ability to modulate the fluidity of cell membranes (173). In keeping, the treatment of breast cancer cell lines with some of these anti-metastatic agents (i.e., alprostadil, amitriptyline, haloperidol, and maprotiline, as well as, salinomycin and thioridazine) reduced membrane fluidity, resulting in EMT impairment, decreased cell motility, and stem cell-like properties, culminating in the impairment of spontaneous metastasis in animal models, thus validating the *in silico* analysis. The impact of fluidity on the metastatic behavior and the strict correlation between membrane fluidity and cholesterol content was further supported by the finding that in breast cancer patients, the overexpression of the cholesterol efflux channel ABCA1 was associated with increased metastatic success and was revealed in 41% of metastatic tumors (173).

### Role and Targeting of TCA Cycle Intermediates and OXPHOS in EMT

Although the contribution of mitochondria to the pathogenesis of cancer was underestimated for a long time, it is now established that mitochondria have an essential role during tumorigenesis. Indeed, mitochondrial alterations are essential for the metabolic rewiring that characterized a given cancer cells and play a role in a series of additional cellular processes during the development and progression of cancer.

We have discussed in details in the first paragraph how aerobic glycolysis is extensively exploited by rapidly proliferating cancer cells to meet their energetic demands and to accumulate biosynthetic precursors. However, several reports support the opposite notion, i.e., that the metabolic requirements of invasive and metastatic cancer cells are characterized by enhanced mitochondrial respiration and ATP generation. This review does not discuss whether and what are the circumstances in which glycolysis or OXPHOS are exploited by cancer cells for promoting their survival and distant colonization. However, we would like to emphasize that contradictory results described the metabolic phenotype of invasive EMT-committed cancer cells as glycolytic or OXPHOS addicted, not only in various tumor types, but also within individual cancer types. As confirmed by clinical analysis of human invasive breast cancers, the transcription coactivator PGC-1α is overexpressed in invasive cancer cells and stimulates mitochondrial biogenesis and OXPHOS during their transit to metastatic sites (153). PGC-1α silencing in cancer cells significantly impaired their invasion ability and decreased the frequency of metastasis without affecting cell proliferation and primary tumor growth. Notably, the PGC-1α-induced metabolic conversion toward OXPHOS is synergistically coupled to a functional EMT program and although PGC-1α-induced pathways are not essential for cancer cells during the acquisition of a mesenchymal phenotype, both pathways concur and correlate with the achievement of invasive and metastatic properties (153).

Accordingly, in both mouse melanoma and human breast cancer models, an overloading of the mitochondrial electron transport chain (ETC) was reported to promote an invasive tumor phenotype associated with increased mitochondrial reactive oxygen species (mtROS) generation (174, 175). However, this study underlined that a dysfunctional mitochondrial activity and a partial lowering of ETC activity strictly resembled ETC overloading, generating a similar pro-oxidant mitochondrial *milieu*. In both cases, superoxide generation induced mitochondrial Src activation, which enhanced the expression of Pyk2, a FAK family member protein tyrosine kinase that was previously reported to promote EMT and migratory abilities (174, 176). Targeting mtROS generation by treating with inhibitors of the ETC Complex I activity (Ebselen) or using specific mitochondria-targeted antioxidants [MitoTEMPO or MitoQ, a mitochondria-targeted form of coenzyme Q10 currently tested in clinical trials (177)] was sufficient to abolish metastasis formation *in vivo* (174).

Although mitochondrial superoxide acts as an inducer/sustainer of EMT, an uncontrolled production of this pro-oxidant can shift cell fate toward senescence or cell death (178, 179). To maintain ROS generation to levels that allow EMT execution, mitochondrial superoxide dismutase 2 (SOD2) have been found upregulated upon TGF-β-mediated EMT, prompting the conversion toward a CSC-like phenotype during the early stage of EMT (180). ZEB2, but not ZEB1, together with NF-κB seems to control the transcriptional regulation of *SOD2*. In addition, a positive feedback loop has been described between SOD2 and NF-κB during EMT in lung adenocarcinoma cells: NF-κB can promote SOD2 transcriptional activation and concomitantly SOD2 induces EMT activating the axis NF-κB/IκB kinase β (IKKβ) (181).

Since an overloading of the respiratory mitochondrial metabolism is often associated with EMT and with the acquisition of pro-invasive skills by cancer cells, drugs that inhibit OXPHOS may be proposed as effective strategies to cope with the acquisition of a motile mesenchymal phenotype endowed with CSC-like features and metastatic competency. Arsenic trioxide, which interfere with OXPHOS by inhibiting the complex III of ETC and metformin, acting on complex I are FDA-approved treatments already used in the clinical practice (182, 183). Crucially, arsenic trioxide has been proposed for the clinical management of relapsed or refractory acute promyelocytic leukemia (183), while metformin particularly exerts anticancer activity in patients with breast, endometrial, or prostate cancer (184, 185).

However, a recent study reported data that partially disagree with the preeminent role of OXPHOS for metastatic potential of cancer cells. In this study, the authors compared mRNA expression of metabolic genes in 20 different solid cancers to investigate the link between metabolic transformation of cancer cells and patient prognosis (186). Data revealed that the inhibition of mitochondrial metabolism is a common signature for all the cancer samples. The downregulation of OXPHOS-related genes correlates with metastatic potential and poor clinical outcome across several cancer types and it is associated with the presence of EMT (186). These results support previous finding that partial mitochondrial dysfunction increases metastatic potential of cancer cells (174) and encourage future investigations on the role of dysregulated metabolism during tumor progression, in order to suggest more suitable targets for clinical intervention.

One of the main metabolic pathways active within mitochondria is the TCA cycle. Numerous enzymes of the TCA cycle show mutation or deregulated expression pattern in both sporadic and hereditary cancers. These alterations identify a subset of patients characterized by poor prognosis (187, 188) and sometimes specific alterations of these mitochondrial enzymes are directly linked to EMT induction.

Consistent with the frequent metabolic rewiring toward OXPHOS observed in invasive cancer cells, epithelial cells that have activated the EMT program may increase the amount of glucose diverted into the TCA cycle by regulating the pyruvate dehydrogenase (PDH) complex, the gatekeeper of the cycle. In particular, in lung cancer cells the execution of the EMT program is accompanied by a downregulation of pyruvate dehydrogenase kinase 4 (PDK4) expression levels, which results in an increase of PDH activity (189). Downregulation of PDK4 is sufficient to drive EMT and promotes erlotinib resistance in EGFR mutant lung cancer cells. In keeping, analysis of human lung adenoma tumor samples reveals PDK4 suppression as a predictor of poor prognosis, consistent with its role during EMT (189).

Besides the deregulation of the expression, specific cancer-associated mutations of some enzymes of the TCA cycle, associated with an alteration of their catalytic function, have been also recognized. Among the mutated enzymes, the most characterized are those involved in the generation of the so-called oncometabolites, including isocitrate dehydrogenase (IDH) (190), succinate dehydrogenase (SDH) (191), and fumarate hydratase (FH) (191).

Isocitrate dehydrogenase catalyzes the reversible conversion of isocitrate into α-KG. Three isoforms of the enzymes have been identified: the NADPH-dependent IDH1 and IDH2 and the NADH-dependent IDH3. Mutations in the cytoplasmic IDH1 and in the mitochondrial IDH2 mutations occur in various human cancers, including gliomas, glioblastoma, and acute myelogenous leukemias (AMLs) (192–194). The mutated forms of the enzymes reduce α-KG into the d-2-hydroxyglutarate (d-2HG). d-2HG levels are low in normal tissues but can reach higher concentration levels (up to millimolar) in IDH1 or IDH2-mutated tumors. High levels of d-2HG interfere with the function of α-KG-dependent dioxygenases, including prolyl hydroxylases (PHDs), essential for the regulation of the stability of HIFs, Jumonji family of histone demethylases, and TET family of DNA demethylases, whose inhibition inevitably impacts on the epigenetic control of gene expression (195, 196). High d-2HG levels paralleled by an altered epigenetic fingerprint was also described in breast tumors characterized by c-Myc overexpression and subsequent metabolic reprogramming, independently of IDH mutations (197). The enantiomer l-2HG (L2HG) can be generated by the non-canonical activity of malate dehydrogenase and LDH and not by the IDH1-/IDH2-mutated enzymes (198, 199). Recently, in colorectal cancer cells an elevation of both d-2HG and its enantiomer l-2HG has been observed in the absence of IDH mutations and it is promoted by glutamine anaplerosis. It has been reported that only d-2HG is able to increase the histone H3 methylation pattern of the ZEB1 promoter region, resulting in a direct upregulation of ZEB1 and downregulation of miR-200, two key drivers of the EMT process (200, 201). Clinical specimens with higher levels of d-2HG associate with colonization of distant organs, supporting the significant contribution of this metabolite in cancer metastasis. Treatment strategies designed to reduce the levels of d-2HG or inhibiting its downstream effects could be effective in colorectal cancer. Interestingly, 2HG has been reported as one of the few metabolites whose levels are reduced upon aspirin administration, currently the most effective drug available for chemoprevention of colorectal cancer (202), suggesting d-2HG as a potential target for therapeutic intervention, with low risk of side effects, since the physiological role of this metabolite is not known. IDH-mutated tumors represent the perfect scenario to test the specific targeting of tumor metabolism with minimal interference with that of normal cells. Inhibitors of the mutant form of IDH have been tested glioma and AML patients harboring IDH mutations (202).

The other two oncometabolites that exert their effects outside of the conventional metabolic network are succinate and fumarate, two TCA cycle intermediates which reach elevated concentrations in some tumors as a consequence of loss-of-function mutations in the SDH complex or the FH, respectively (203–205). Like d-2HG, succinate and fumarate have been found to interfere with dioxygenase activity, underlining the oncogenic role of both these metabolites in the inhibition of PHDs and the subsequent stabilization of HIF-1 (206) and supporting the notion that a general property of oncometabolites is the ability to regulate epigenetics (191, 207).

Interestingly, fumarate possesses another unique property linked to its chemical structure. Indeed, fumarate can covalently bind to cysteine residues of proteins in a process called succination (208, 209). Several proteins are succinated in FH-deficient cells, including aconitase (210), Kelch-like ECH-associated protein 1 (208, 209), and glutathione (211). Notably, in a subset of FH-deficient human renal cell carcinomas, fumarate at high concentration directly bounds the antioxidant glutathione both *in vitro* and *in vivo* to produce the metabolite succinated glutathione (211). Succinated glutathione acts as an alternative substrate to glutathione reductase to decrease NADPH levels and enhance mtROS and HIF-1 activation, two mandatory events fostering EMT execution.

More recently, Sciacovelli and colleagues demonstrate a fascinating role of fumarate, which accumulates in FH-deficient renal cancers, in the epigenetic suppression of miR-200 through the inhibition of Tet-mediated demethylation of a regulatory region of the anti-metastatic miRNA cluster *mir-200ba429* (212). Fumarate-dependent miR-200 downregulation leads to the expression of EMT-related transcription factors and enhanced aggressive features and it is associated with a poor clinical outcome (212). Although it has been extensively reported that FH deficiency is associated to fumarate-dependent epigenetic deregulation, supporting the notion of FH as a tumor suppressor, the molecular mechanisms by which FH gene expression is controlled is not well clarified. It has been recently reported that in nasopharyngeal carcinoma, the chromatin remodeling factor lymphoid-specific helicase binds the FH promoter and recruits the epigenetic silencer factor G9a to inhibit the transcription of FH (213). The FH reduction promoted an unbalance in the TCA intermediates, with a decrease in malate levels and a concomitant increase in αKG amount. Deregulation of TCA metabolites in nasopharyngeal carcinoma cells induces the recruitment of IKKα to the promoter regions of genes involved in the EMT program, causing the downregulation of E-cadherin and ZO-1 and the parallel upregulation of the mesenchymal marker vimentin, leading to EMT, migration and invasion *in vitro*, and increase metastasis *in vivo* (213).

Deregulation of different components of the SDH complex, enzyme involved in both the ETC and the TCA cycle, have been reported in several cancer types (214, 215). SDH is composed of four essential subunits: the flavoprotein SDHA, the iron-sulfur protein SDHB, and two units anchored to the mitochondrial membrane SDHC and SDHD. Mutations in *SDHx* genes (encoding SDH subunits) lead to succinate accumulation that inhibits DNA demethylases (TET enzymes), leading to a global hypermethylation of DNA (191, 216, 217). Crucially, SDHx mutations have been associated with EMT in hereditary pheochromocytoma and paraganglioma, as a result of epigenetic alterations (218, 219). In particular, Aspuria et al. demonstrated that *Sdhb* knockdown in mouse ovarian cancer cells resulted in a global hypermethylation pattern that promotes EMT and induces a metabolic reprogramming of the central carbon metabolism, together with additional mitochondrial dysfunction, ultimately leading to altered glucose and glutamine utilization (220). More recently, Loriot and colleagues clarified how the succinate-mediated epigenetic modulation impacts on EMT. First, using transcriptome profiling of a large cohort of metastatic pheochromocytomas and paragangliomas, the authors reported that *SDHB*-malignant samples displayed a change in the expression pattern of Twist1, Twist2, Snail1, and N-cadherin, all indications of EMT program activation (221). Moreover, cytokeratin 19 (KRT19), a component of the intermediate filaments, already implicated in conferring enhanced migratory and invasive properties in squamous cell carcinomas, neuroblastomas, renal, and breast cancers (222–225), has been identified as one of the most significantly hypermethylated and downregulated gene in *SDHB*-deficient mouse chromaffin cells, supporting the EMT-dependent invasive properties and the metastatic behavior of these neuroendocrine cancer cells (218).

Besides the four subunits that constitute the SDH complex, SDH5 has been identified as a mitochondrial protein necessary for the flavination of SDHA and for the assembly of the SDH complex. Additionally, several independent reports pointed to a role of SDH5 as a tumor-suppressor gene and SDH5 deregulated expression correlate with higher tumor incidence (226–228). An interesting study reported that SDH5 loss in lung cancers promotes tumor aggressiveness and metastasis, hence providing an additional functional support on the link between SDH5 and EMT. In particular, SDH5 acts as a modulator of the glycogen synthase kinase 3β (GSK-3β)/β-catenin signaling, since the physical interaction between SDH5 and GSK-3β induces GSK-3β dephosphorylation/activation, the subsequent decrease of β-catenin nuclear accumulation and activation, thereby ultimately inhibiting Wnt-β-catenin signaling (229). The downregulation of SDH5 observed in lung cancers accounts for the SDH5-mediated β-catenin stabilization and transcriptional activity, which in turn induces Slug and Twist1 gene expression, thereby repressing E-cadherin and contributing to EMT (229).

Besides SDH5 repression, other mitochondrial signaling pathways concur to regulate the GSK-3β/β-catenin pathway, impacting on EMT. In lung cancer cells, the downregulation of the TU translation elongation factor mitochondrial (TUFM), a key factor in the translational expression of mitochondrial DNA, is associated to the maintenance of the mesenchymal phenotype of cancer cells and to the acquisition of more aggressive features. TUFM downregulation results in a reduced expression of mtDNA, leading to mitochondrial dysfunction and cellular stresses such as ATP deficiency and ROS production, which trigger AMPK activation. It has been reported that activated AMPK induces the phosphorylation of GSK-3β and increases the nuclear accumulation of β-catenin, leading to the induction of EMT and increased migration and metastatic competency of lung cancer cells (230). These results provide a molecular link between mitochondrial dysfunction and EMT, which is implicated in lung cancer progression.

Malic enzyme 1 (ME1) is reported to be a major metabolic enzyme, both localized in cytosol and mitochondria, catalyzing the oxidative decarboxylation of malate into pyruvate, thus fueling the TCA cycle, and concomitantly reducing NADP^+^ into NADPH and contributing to macromolecular biosynthesis and protection from excessive oxidative stress. For its essential metabolic roles, ME1 is ubiquitously expressed in different human tissues. However, up to now, there are no evidence about its oncological functions and clinic significance. A significant upregulation of ME1 has been associated to aggressive hepatocellular carcinoma and to reduced overall survival and progression-free survival in this tumor type (231). Silencing of ME1 in hepatocellular carcinoma cells inhibits migration and invasion by blocking the EMT program (i.e., restoring E-cadherin while downregulating N-cadherin and vimentin expression) in a ROS-dependent way, suggesting ME1 as a poor prognostic predictor for hepatocellular carcinoma-bearing patients (231).

## Concluding Remarks

Epithelial-to-mesenchymal transition is a specific program that confers the cancer cell a series of stem-like properties and migratory abilities thus increasing cancer aggressiveness. The hostile microenvironment (i.e., hypoxia, low pH, and low glucose) and the metabolic requirements of a fast-growing tumor sustain EMT execution allowing cancer cells to bypass the nutrients and oxygen supply limitation caused by the rapid primary cancer growth and colonize secondary sites to secure the adequate support of energy and nutrients. However, this is not simply a process to acquire migration and invasion abilities but a more complex rewiring of signaling, metabolic, genetic, and epigenetic networks that allow differentiated epithelial cancer cells to revert into a more undifferentiated state and stem cell functions. Additionally, it is plausible to speculate that aggressive cancer cells are characterized by the ability to undergoing EMT, acquire stem cell-like traits, and rewire their metabolism to gain a plethora of strategies to survive in different environmental conditions and/or in presence of anticancer drugs. This plasticity reinforces the conclusion that “*it is not the fittest of the species (in this case of the tumor cell bulk) that survives but the most adaptable*.” The challenges we now face are (i) the identification of distinct metabolic hallmarks exclusive to cancer-associated EMT and (ii) the identification of therapeutic window that allows EMT targeting in cancer patients. These are key questions that need to be addressed to reduce the off target effects caused by general metabolic therapies and importantly to avoid the administration of anti-EMT compounds to cause a MET in cells that have already left the primary site and may give rise to metastases. However, none of the clinical trials using anti metabolic drugs, extensively reviewed in Ref. (6), have investigated whether the effects exerted by these compounds have an impact on the EMT process and therefore further confirmation in preclinical studies and the design of large prospective clinical trials should be planned.

## Author Contributions

AM, MT, PC, and EG were responsible for the conception, design and drafting of the article, and final approval; agreed to be accountable for all aspects of the work in ensuring that questions related to the accuracy or integrity of any part of the work are appropriately investigated and resolved.

## Conflict of Interest Statement

The authors declare that the research was conducted in the absence of any commercial or financial relationships that could be construed as a potential conflict of interest. The reviewer MB and handling editor declared their shared affiliation, and the handling editor states that the process nevertheless met the standards of a fair and objective review.

## Abbreviations

ACC, acetyl-CoA carboxylase; ACSLs, acyl-CoA synthetases; AMF, autocrine motility factor; CAFs, cancer-associated fibroblasts; CAIX, carbonic anhydrase IX; CerS6, ceramide synthase 6; CHC, α-cyano-4-hydroxycinnamate; ChREBP, carbohydrate-responsive element-binding protein; CSC, cancer stem cell; DHA, docosahexaenoic acid; ECM, extracellular matrix; EMT, epithelial-to-mesenchymal transition; ETC, electron transport chain; FAs, fatty acids; FASN, fatty acid synthase; FBP1, fructose-1,6-bisphosphatase; FH, fumarate hydratase; GDH, glutamate dehydrogenase; GLS, glutaminase; GLUT, glucose transporter; GSK-3β, glycogen synthase kinase 3β; d-2HG, d-2-hydroxyglutarate; HGF, hepatocyte growth factor; HIF-1α, hypoxia-inducible factor-1α; HK2, hexokinase; IDH, isocitrate dehydrogenase; IL-8, interleukin-8; α-KG, α-ketoglutarate; LDs, lipid droplets; LDH, lactate dehydrogenase; MCTs, monocarboxylate transporters; ME1, malic enzyme 1; MET, mesenchymal-to-epithelial transition; MMPs, matrix metalloproteases; mtROS, mitochondrial reactive oxygen species; NHE1, sodium-proton exchange 1; NSCLC, non-small cell lung cancer; *O*-GlcNAc, *O*-linked *N*-acetyl-glucosamine; OXPHOS, oxidative phosphorylation; PDAC, pancreatic ductal adenocarcinoma; PDH, pyruvate dehydrogenase; PDK4, pyruvate dehydrogenase kinase 4; PGI, glucose phosphate isomerase; PM, plasma membrane; PK, pyruvate kinase; PPARGC1A/PGC-1α, peroxisome proliferator-activated receptor gamma coactivator 1 alpha; PPP, pentose phosphate pathway; SAH, S-adenosyl homocysteine; SAM, S-adenosyl methionine; SCD, stearoyl-CoA desaturase-1; SDH, succinate dehydrogenase; SIRT, sirtuin; SOD2, superoxide dismutase 2; SREBPs, sterol regulatory element-binding proteins; TAGs, triacylglicerols; TCA, tricarboxylic acid; TDH, threonine dehydrogenase; TET1, ten–eleven translocation 1; TGF-β, transforming growth factor-β; TGIF2, TGF-β-induced factor homeobox 2; TKTL1, transketolase-like protein 1 isoform; TNBC, triple-negative breast cancer; TNF-α, tumor necrosis factor-α; TUFM, TU translation elongation factor mitochondrial; VEGF, vascular endothelial growth factor.
